# Primary pleural liposarcoma combined spindle cell lipoma of the lung

**DOI:** 10.1111/1759-7714.13495

**Published:** 2020-05-21

**Authors:** Lae Hyung Kang, Chung Su Hwang, Seong Hoon Yoon

**Affiliations:** ^1^ Department of Internal Medicine, School of Medicine Pusan National University Yangsan Republic of Korea; ^2^ Department of Pathology, School of Medicine Pusan National University Yangsan Republic of Korea

**Keywords:** Liposarcoma, MDM2, spindle cell lipoma

## Abstract

Liposarcoma is a malignant adipose tissue tumor which mainly originates from the extremities and retroperitoneum. Primary pleural liposarcoma is very rare. Spindle cell lipoma is a rare benign adipose tissue tumor. A 66‐year‐old male was referred to our hospital for the evaluation of a mass‐like opacity visible on chest X‐ray. Computed tomography (CT) scan revealed a well‐defined soft tissue mass with internal low attenuations and adjacent multiple nodules in the upper lobe of the left lung, and surgical excision was subsequently performed. Histopathological findings revealed adipose tissue with lipoblasts and spindle cells and immunohistochemical staining (IHC) revealed the tumor cells were strongly positive for CDK4 and MDM2. Histopathological examination of the small lung nodules showed spindle cell proliferation and adipose tissue without positivity for MDM2. Here, we report a rare case of primary pleural liposarcoma combined adjacent spindle cell lipoma of the lung.

## Introduction

Liposarcomas are soft tissue sarcomas that usually present in the extremities and retroperitoneum. Primary pleural liposarcoma is extremely rare and more common in males.^1^ Spindle cell lipoma, which often occurs in the neck and back of middle‐aged men, is a rare benign adipose tissue tumor.^2^ Spindle cell lipoma of the lung is usually seen as multiple nodules on computed tomography (CT) and is also very rare. The diagnosis of these lipomatous tumors is usually based on pathological examination via surgical excision. Here, we describe a case of primary pleural liposarcoma combined spindle cell lipoma of the lung confirmed following surgical excision.

## Case report

**Figure 1:**
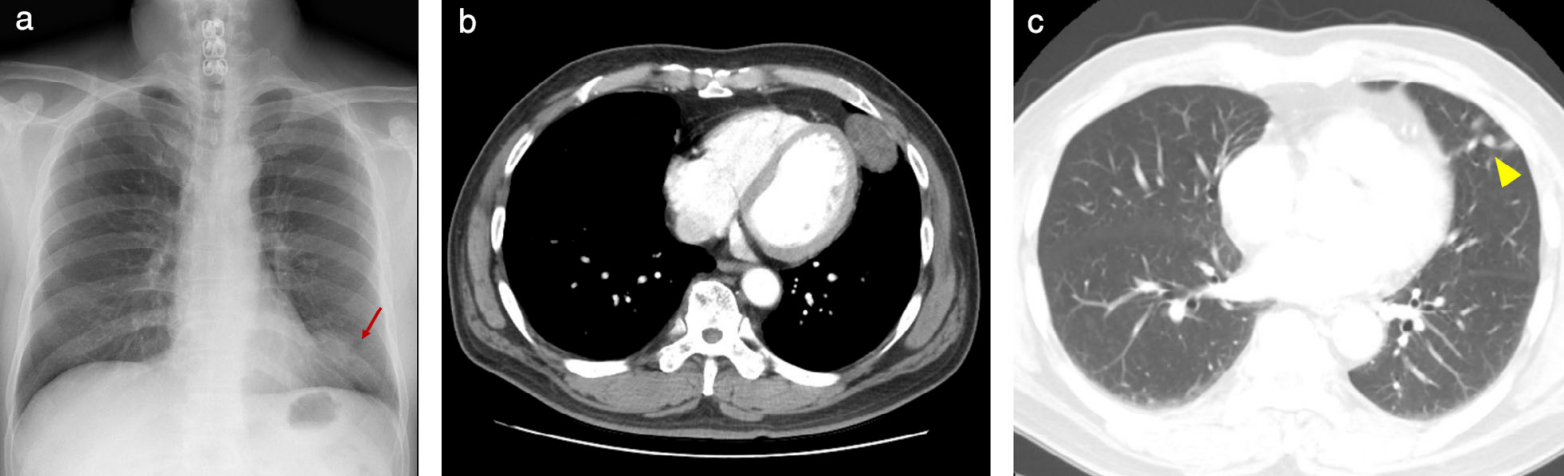
Chest images. (**a**) Chest X‐ray revealed a mass‐like opacity in the lower zone of the left lung (arrow). (**b**) Computed tomography (CT) scan of the chest showed an inhomogenous well‐defined mass with internal low attenuations in the left lower paracardiac area. (**c**) CT scan of the chest showed multiple subpleural nodules in the upper lobe of the left lung (arrowhead).

**Figure 2:**
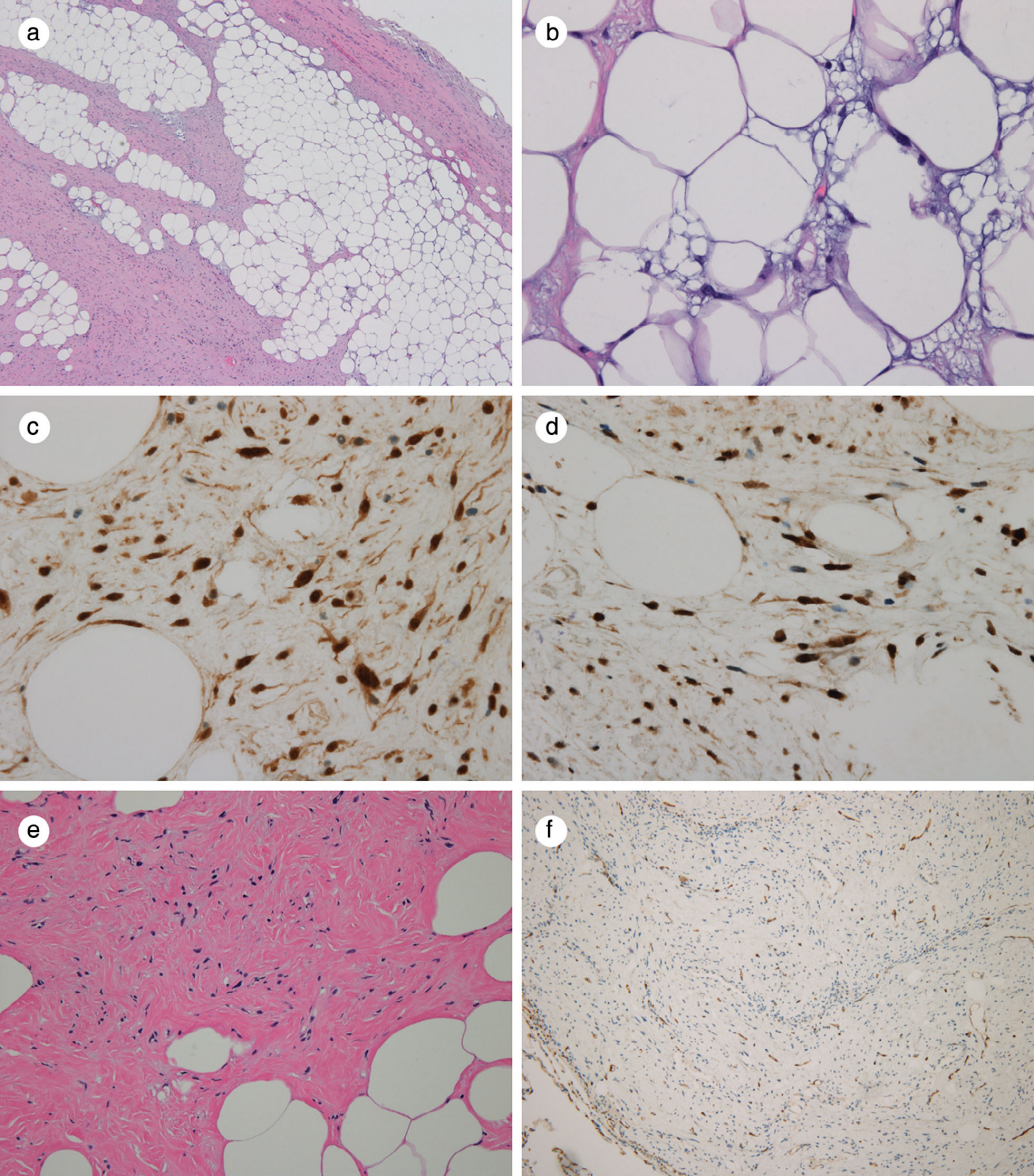
Histopathological images. (**a**) The tumor consisted of adipose tissue and a fibrotic area (hematoxylin and eosin staining, ×40). (**b**) Some areas of adipose tissue showed atypical lipoblasts with atypical nuclei and multiple fat vacuoles (hematoxylin and eosin staining, ×400). (**c**) Nuclei of atypical spindle cells and atypical lipoblasts were strongly positive for CDK4 (×400). (**d**) Immunohistochemical staining showed diffuse and strongly positivity for MDM2 (×400). (**e**) The tumor was composed of hypocellular spindle cell proliferation and adipose tissue (hematoxylin and eosin staining, ×200). (**f**) Immunohistochemical staining revealed that CD34 was focally positive (×100).

A 66‐year‐old male was referred to our hospital for further evaluation of a lung mass, which was incidentally found on CT scan. He was an ex‐smoker and had no previous medical history. Physical examination showed no abnormalities in the chest and revealed stable vital signs. Chest X‐ray showed a mass‐like opacity in the lower zone of the left lung (Fig 1). CT scan of the chest revealed a well‐defined oval mass with internal low attenuation measuring 4.2 × 2.5 cm in size at the left upper lobe of the lung with adjacent separate nodules (Fig 1). Percutaneous needle biopsy guided by CT scan was first performed, but not enough specimen could be obtained for pathological confirmation. The patient subsequently underwent mass excision via video‐assisted thoracoscopic surgery (VATS). At surgery, the mass appeared to originate from the pleura. Grossly, the excised tumor was a well‐defined yellowish solid mass that measured 4.5 × 3.3 × 3.0 cm in dimension. The resected lung specimen contained three ill‐defined yellow‐white colored solid nodules measuring 0.7 × 0.6 cm, 0.6 × 0.6 cm, and 0.5 × 0.5 cm in size, respectively. Histopathological findings of the pleural mass showed adipose tissue with atypical lipoblasts and irregular fibrous septa that consisted of spindle cells without mitotic figures (Fig 2). Immunohistochemical staining (IHC) revealed the tumor cells were strongly positive for CDK4, and MDM2 (Fig 2). Well‐differentiated pleural liposarcoma was confirmed on the basis of these findings. Histopathological examination of the lung specimen revealed three nodules consisting of benign and hypocellular spindle cell proliferation admixed with adipose tissue and collagen fibers that was negative for MDM2 on immunohistochemical staining (Fig 2). These findings were compatible with a diagnosis of spindle cell lipoma.

## Discussion

Liposarcoma accounts for about 20% of all mesenchymal tumors, is the most common soft tissue sarcoma in adults and is usually found in the retroperitoneum and the extremities.^1^ Primary intrathoracic liposarcomas are rare, representing 2.7% of all locations and can occur in the lung, mediastinum, pleura, and chest wall.^3^ Most malignant tumors originating from the pleura are metastatic, while primary pleural tumors such as malignant mesothelioma, fibrous tumor and pleural liposarcoma account for approximately 10% of them.^4^

Primary pleural liposarcoma is extremely rare and the exact incidence is unknown. It is known to be more common in males and is primarily a disease of individuals of 50 years of age.^5^ It tends to remain clinically asymptomatic due to indolent features and is incidentally found on lung imaging as was the case with our patient. However, it may lead to nonspecific symptoms such as chest pain, cough, and dyspnea because of displacement and compression of adjacent structures.^6^ Imaging evaluation such as CT scan and magnetic resonance imaging (MRI), which usually reveal a hypodense mass with fat component, is useful to determine the extent of tumor invasion and the resectability. Surgical resection is recommended as the standard of care and adjuvant chemoradiotherapy can be given as appropriate.^7^

Liposarcomas originate from the primitive mesenchymal tissue residues rather than mature fat tissue. While the pleural cavity is formed from mesoderm, mesenchymal cells that are left in this process go through malignant transformation and bring about pleural liposarcoma.^8^ Liposarcomas are divided into well‐differentiated, myxoid, pleomorphic, and dedifferentiated subtypes reflecting their complex histological components.^9^ Our case was consistent with the well‐differentiated subtype, which is the most common with the best prognosis. Well differentiated liposarcoma is characterized by amplification of chromosome 12q13‐15, which carries the oncogenes *MDM2*, *CDK4*, and *HMGA2*.^10^

Spindle cell lipoma, which is composed of mature adipose tissue, collagen, and spindle cells is a rare benign tumor and commonly found in middle‐aged and older males.^2^ Most cases present as a subcutaneous mass in the neck, back, and proximal upper extremity, but some cases are found in atypical locations such as the thoracic cavity.^11^ Intrathoracic spindle cell lipoma is mostly asymptomatic and has no specific imaging findings. The most common imaging appearance is a tumor than contains fat and the correct preoperative diagnosis is difficult.^12^ Surgical excision is recommended as the treatment of choice for spindle cell lipoma.

Intrathoracic spindle cell lipoma is usually diagnosed by histological examination. Microscopic appearance of spindle cell lipoma is characterized by the classic triad of mature adipocytes, ropey collagen bundles, and bland spindle cells. Spindle cell lipoma is strongly CD34 positive on immunohistochemical staining and the spindle cells may stain for S‐100.^13^ MDM2 immunohistochemistry can be used to differentiate lipoma from liposarcoma, which is mostly positive for MDM2.^13^ In our case, spindle cell lipoma was confirmed on the basis of typical morphology, negative MDM2 test, and CD34 positivity.

In conclusion, here we report a rare case of primary pleural liposarcoma combined spindle cell lipoma of the lung. This report might be helpful for the diagnosis of rare intrathoracic lipomatous tumors in the future.

## Disclosure

The authors declare that there are no conflicts of interest.
